# Spectrophores as one-dimensional descriptors calculated from three-dimensional atomic properties: applications ranging from scaffold hopping to multi-target virtual screening

**DOI:** 10.1186/s13321-018-0268-9

**Published:** 2018-03-07

**Authors:** Rafaela Gladysz, Fabio Mendes Dos Santos, Wilfried Langenaeker, Gert Thijs, Koen Augustyns, Hans De Winter

**Affiliations:** 1 Laboratory of Medicinal Chemistry, Department of Pharmaceutical Sciences, Faculty of Pharmaceutical, Biomedical and Veterinary Sciences, Campus Drie Eiken, Building A, Universiteitsplein 1, 2610 Antwerp, Belgium; 2 Department of Chemistry, Faculty of Science, Campus Diepenbeek, Agoralaan, Building D, 3590 Diepenbeek, Belgium; 3 Agilent, Clinical Applications Division, Technologielaan 3, 3001 Louvain, Belgium

**Keywords:** Spectrophore, Pharmacophore, Affinity fingerprinting, Artificial cage, Descriptor, Fingerprint, Compound similarity, QSAR, Tanimoto, Acetylcholinesterase, Thrombin, Scaffold hopping, Virtual screening

## Abstract

Spectrophores are novel descriptors that are calculated from the three-dimensional atomic properties of molecules. In our current implementation, the atomic properties that were used to calculate spectrophores include atomic partial charges, atomic lipophilicity indices, atomic shape deviations and atomic softness properties. This approach can easily be widened to also include additional atomic properties. Our novel methodology finds its roots in the experimental affinity fingerprinting technology developed in the 1990’s by Terrapin Technologies. Here we have translated it into a purely virtual approach using artificial affinity cages and a simplified metric to calculate the interaction between these cages and the atomic properties. A typical spectrophore consists of a vector of 48 real numbers. This makes it highly suitable for the calculation of a wide range of similarity measures for use in virtual screening and for the investigation of quantitative structure–activity relationships in combination with advanced statistical approaches such as self-organizing maps, support vector machines and neural networks. In our present report we demonstrate the applicability of our novel methodology for scaffold hopping as well as virtual screening.

**Electronic supplementary material:**

The online version of this article (10.1186/s13321-018-0268-9) contains supplementary material, which is available to authorized users.

## Introduction

Computational drug design has played a major role in the discovery of molecular therapeutics for more than three decades. This domain can be broadly classified into protein structure-based and ligand-based methods. Protein structure-based methods rely on the availability of structural information of both protein target and ligands, and includes technologies such as computational fragment-based drug design [1] and molecular docking [2, 3]. Ligand-based methods use only information obtained from the ligands for predicting activity, dependent on their similarity or dissimilarity to previously known active ligands. Widely used ligand-based methods include pharmacophore searching [4, 5], bitwise fingerprint-based similarity searches [6–9] and the development of quantitative structure–activity/property relationships involving a variety of different molecular descriptors [10–13]. Additionally, important cheminformatics approaches such as the establishment and maintenance of compound databases [14–17], compound clustering [18, 19] and maximum common substructure calculations [20, 21] are now firmly integrated into the workflow of many pharmaceutical drug discovery processes.

Ligand-based virtual screening approaches all rely on the concept that structurally similar molecules have similar biological activities [22]. Molecular fingerprints are bitwise representations of molecular structure and properties and examples include hashed connectivity pathways [23], dictionary-based [24], and layered atom fingerprints [25, 26]. Another example are the 3D-MoRSE descriptors [27]. These methods are also called two-dimensional (2D) similarity methods since these do not rely on the underlying three-dimensional (3D) structure of the molecules.

It has been shown that in a significant number of cases ligand-based virtual screening outperforms protein structure-based virtual screening [28], although the latter performs better in scenarios where novel scaffolds need to be identified [29]. 3D similarity virtual screening methods make use of the three-dimensional structure of the reference compound, as a query to search for compounds that have similar spatial atomic arrangements. These methods are not dependent upon the underlying molecular topology of the query compounds and are therefore also useful for scaffold hopping. Examples of such algorithms include shape-matching algorithms and shape-based fingerprints [30, 31], molecular field descriptors [32, 33], pharmacophore fingerprints [34–36] and pharmacophore-based screening [37–39]. A number of recent reviews on the use of descriptors and classification methods are available [40–43].

In this study, a novel shape-based descriptor is described which is termed a ‘spectrophore’, referring to the fact that this descriptor is composed of a one-dimensional ‘spectrum’ of *n* real numbers, with each of these numbers representing the interaction between a given molecular property and a certain artificial environment (hence resembling a type of ‘pharmacophore’). Because spectrophores are shape-based, these descriptors are not directly dependent on the actual molecular topology but rather on the molecular field that is generated by the underlying topology, hence craving it use as a scaffold-hopping tool in combination with automated molecular design approaches. In addition, since the spectrophores are composed of a set of real numbers and being independent on the underlying molecular orientation, these descriptors can be used as input to automated machine learning approaches for the generation of advanced QSAR models. The spectrophore approach has been based on the affinity fingerprinting technology, which was originally described in the 90’s by Terrapin Technologies, Inc. [44]. In this approach, an affinity fingerprint is the pattern of the in vitro binding potency of a single compound to a reference panel of eight diverse proteins. Using a database of such affinity fingerprints, the authors were able to predict the binding potency of a novel compound for a specific protein target using a multivariate linear regression model, derived from the affinity fingerprints of a small set of training compounds. An analogous fingerprinting system is used in the spectrophore technology presented here, but in which the diverse set of reference proteins of the original affinity fingerprinting technology has been replaced by a set of virtual affinity cages. In addition, the in vitro measured binding potencies have also been replaced by the calculated interaction energies between a number of atomic properties and the surrounding cage points. We report the applicability of this approach for virtual screening and compound clustering. The influence of the conformational flexibility on the generated spectrophores is also discussed.

## Method

### Artificial cages surrounding the molecule

**Fig. 1:**
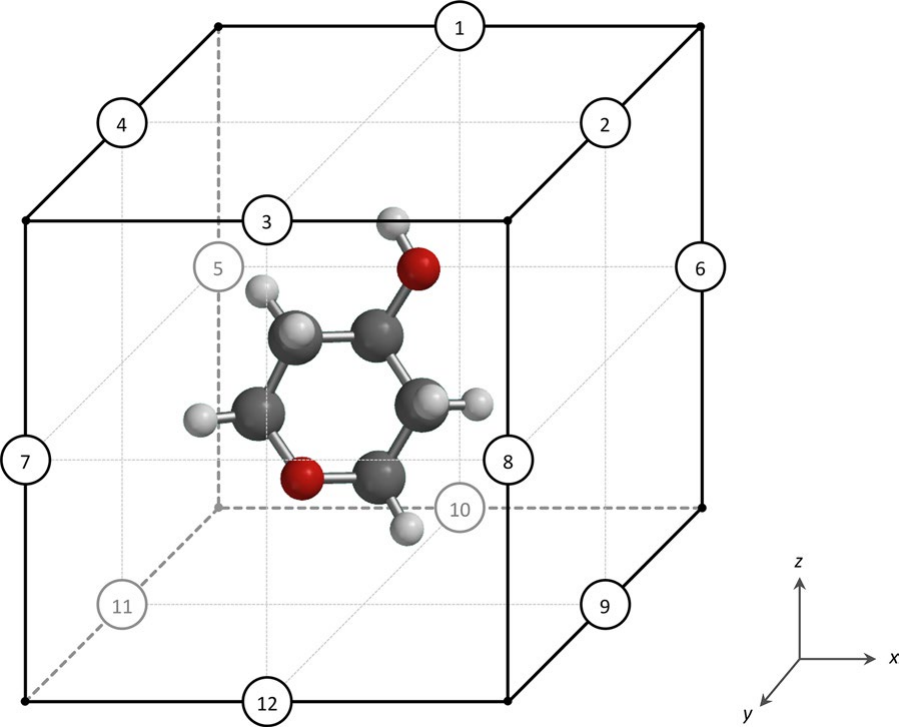
Schematic representation of the artificial cage with the 12 points labeled. Each point is assigned a value of + 1 or − 1, with the constraint that the sum of all values must be zero. A molecule enclosed by the cage is also shown

Spectrophores are generated by calculating the interaction energies between the molecule and a set of predefined artificial cages that surround the molecular conformation. Each cage consists of 12 points and each point is assigned a value of + 1 or − 1, with the additional constraint that the sum of the values on all points on the cage should be 0 (hence each cage consists of six points with value + 1 and six points with value − 1) (Fig. 1).

Within the constraint that the sum of all values on the cage must be zero, it is possible to construct either 12 or 18 unique cages, depending on whether the + 1 and − 1 values are distributed in either a symmetrical or asymmetrical manner along the cage. These different cages are summarized in Table 1.

**Table 1:** Distribution of + 1 and − 1 values on each of the cages

Non-stereospecific cages	Grid points											
	1	2	3	4	5	6	7	8	9	10	11	12
Cage 1:	+	+	–	–	–	+	+	–	–	–	+	+
Cage 2:	+	+	–	–	+	–	–	+	–	–	+	+
Cage 3:	+	+	–	–	+	–	–	+	–	–	+	+
Cage 4:	+	+	+	–	–	–	–	–	+	+	–	+
Cage 5:	+	+	+	–	–	+	–	+	–	–	+	–
Cage 6:	+	+	+	–	+	–	+	–	–	–	+	–
Cage 7:	+	+	+	–	+	–	+	–	+	–	–	–
Cage 8:	+	+	+	+	–	–	–	–	+	–	+	–
Cage 9:	+	+	+	+	–	–	–	–	+	+	–	–
Cage 10:	+	+	+	+	+	–	–	+	–	–	–	–
Cage 11:	+	+	+	+	+	+	–	–	–	–	–	–
Cage 12:	+	+	+	–	–	+	–	–	–	+	–	+

Stereospecific cages	Grid points											
	1	2	3	4	5	6	7	8	9	10	11	12
Cage 1:	+	+	–	–	+	–	+	–	+	–	–	+
Cage 2:	+	+	+	–	–	–	–	–	+	+	+	–
Cage 3:	+	+	+	–	–	+	–	–	–	–	+	+
Cage 4:	+	+	+	–	+	–	–	–	–	+	–	+
Cage 5:	+	+	+	–	+	–	–	–	–	–	+	+
Cage 6:	+	+	+	–	+	–	–	–	+	–	+	–
Cage 7:	+	+	+	–	+	–	–	–	+	–	–	+
Cage 8:	+	+	+	–	+	+	–	–	–	–	–	+
Cage 9:	+	+	+	–	+	+	–	–	+	–	–	–
Cage 10:	+	+	+	–	+	–	–	+	–	+	–	–
Cage 11:	+	+	+	–	+	–	–	+	–	–	+	–
Cage 12:	+	+	+	–	+	–	–	+	–	–	–	+
Cage 13:	+	+	+	–	+	+	–	+	–	–	–	–
Cage 14:	+	+	+	–	+	+	–	–	–	–	+	–
Cage 15:	+	+	+	–	+	–	+	–	–	+	–	–
Cage 16:	+	+	+	–	+	+	+	–	–	–	–	–
Cage 17:	+	+	+	+	+	–	–	–	+	–	–	–
Cage 18:	+	+	+	+	+	–	–	–	–	+	–	–

Grid point numbering refers to the numbering shown in Fig. 1. There are 12 cages with a center of symmetry (hence non-stereospecific cages), and 18 cages without a center of symmetry (stereospecific cages). ‘+’ represents a value of + 1, and ‘−’ represents a value of − 1

Each molecule (or more specific: each conformation) is inserted into each of these cages with the molecular center of geometry corresponding to the center of the cage. The initial orientation of the molecule is taken from the input geometry provided by the user; however this parameter is not important as the molecule is subsequently rotated along all its axes within the surrounding cage (see below). In our current implementation we opted to use a rectangular cage with the cell dimensions adjusted in such a manner that the minimum distance between the enclosed molecule and each of the cell edges corresponds to a constant value which is in the same range of a typical ligand-receptor non-bonded contact, for example around 3 Å, and which can be specified at runtime (corresponds to the resolution of the spectrophore; see below). This constant distance between molecule and cage is established by altering the cage dimensions on each new orientation of the enclosed molecule.

### Atomic properties

**Fig. 2:**
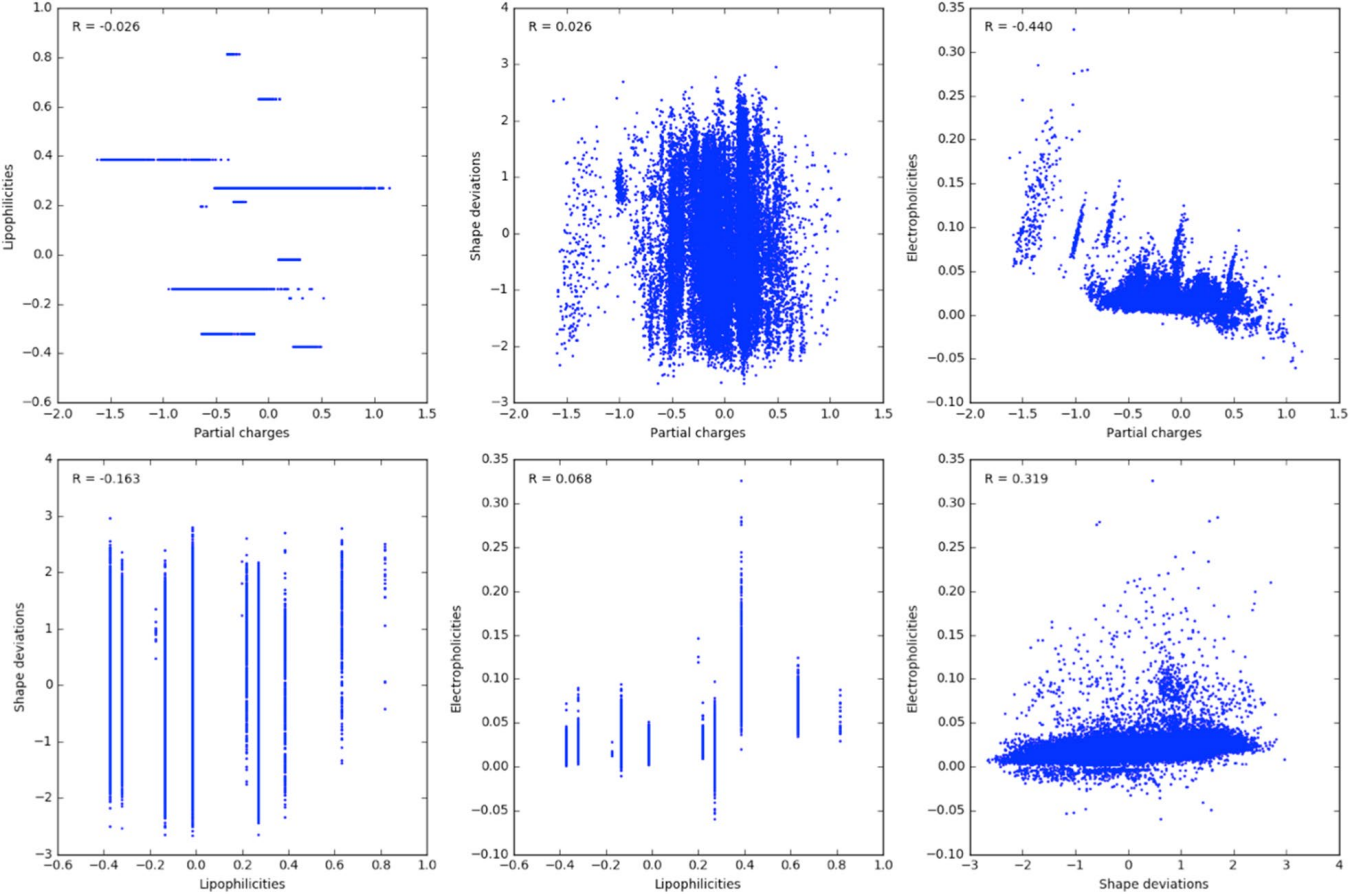
Correlation plots between the four atomic properties that were used to calculate spectrophores. Correlation coefficients are also given for each property pair. Atomic properties were calculated from a randomly selected subset of 10,000 compounds from the DUD-E database [45, 46]. The lipophilicities are discrete since there only 12 different atomic lipophilic properties (see Table 2 below)

The generation of a spectrophore requires the calculation of a number of atomic properties of which the interaction energy with the cage point values is obtained. In our current implementation, four atomic properties were generated, which include the atomic partial charges, atomic lipophilicities, atomic shape deviations and atomic electrophilicities. These properties were selected based on the fact that no or little correlation exists between each of these, as shown in Fig. 2 in which the atomic properties calculated from a subset of 10,000 compounds randomly selected from the DUD-E dataset [45, 46] are plotted against each other.

Atomic shape deviations are generated by calculating, for each atom, the atom’s deviation from the average molecular radius. Atomic partial charges and atomic electrophilicity properties are calculated using the electronegativity equalization method, as described by Bultinck and coworkers [47, 48]. Atomic lipophilicities are assigned using a rule-based approach, according to the particular atom type. Parameters used to calculate lipophilicity, electronegativity and partial charges are summarized in Table 2.

**Table 2:** Parameters used to calculate the atomic partial charges [47, 48], atomic electronegativities [47, 48] and atomic lipophilicities

Atom	χ	η	Atomic lipophilicity
H (polar)	+ 0.206	+ 0.660	− 0.374
H (connected to C or H)	+ 0.206	+ 0.660	− 0.018
Li, B, Na, Mg, Si, P, K, Ca, Fe, Cu, Zn	+ 0.362	+ 0.330	− 0.175
C	+ 0.362	+ 0.330	+ 0.271
N	+ 0.493	+ 0.345	− 0.137
O	+ 0.730	+ 0.544	− 0.321
F	+ 0.721	+ 0.727	+ 0.217
S	+ 0.620	+ 0.206	+ 0.385
Cl	+ 0.362	+ 0.330	+ 0.632
Br	+ 0.701	+ 0.546	+ 0.815
I	+ 0.681	+ 0.307	+ 0.198
Any other element	+ 0.206	+ 0.660	− 0.175

### Interaction energies

**Fig. 3:**
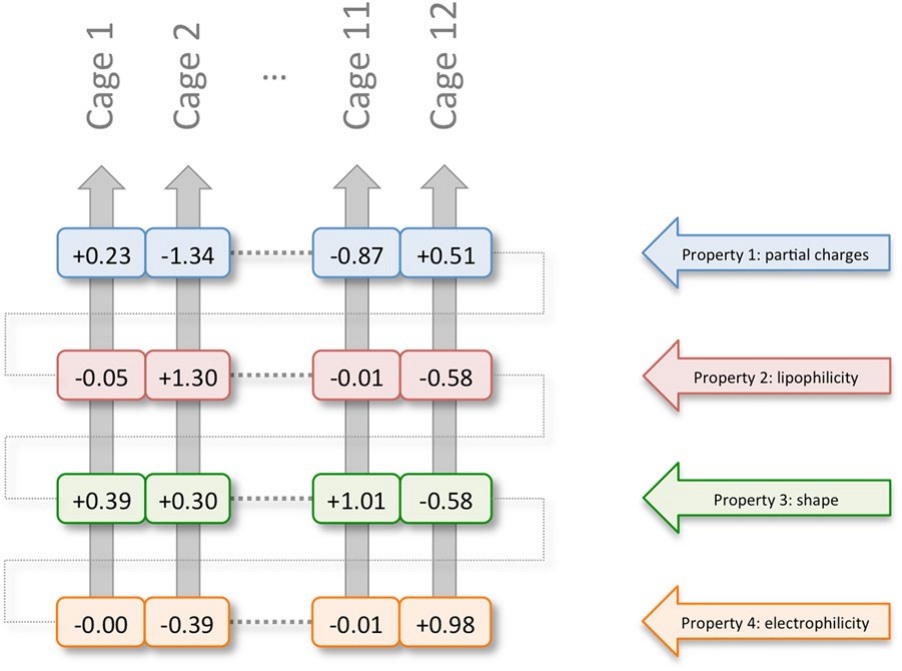
Dissection of the spectrophore vector using a hypothetical example. Each value represents the maximal interaction energy value *V*(*c*, *p*) between property *p* and cage *c* as calculated according Eq. 1, optimized by rotating the molecule in the box and keeping the largest value. Only shown are the spectrophore values calculated with cages 1, 2, 11 and 12

For a molecular conformation with *j* atoms and *p* atomic properties (in the current implementation *p* = 4), the total interaction value *V*(*c*, *p*) between the atomic contribution values *A*(*j*, *p*) of property *p* and the cage values *P*(*c*, *i*) for a given cage *c* with *i* cage points, is calculated according a standard interaction energy equation as given in Eq. 1:

1 $$V(c,p) = - 100\sum\limits_{i} {\sum\limits_{j} {\frac{A(j,p)P(c,i)}{{r_{ij} }}} }$$

with *r*_*ij*_ being the Euclidean distance between cage point *i* and atom *j*. The arbitrary factor of − 100 in the equation above is used to scale the calculated interaction values to a number of order ~ 1, with attractive interaction values expressed as positive numbers and repulsive values as negative numbers. This total interaction energy *V*(*c*, *p*) for a given property *p* and cage *c* is maximized by rotating the molecular orientation along the three angular dimensions and calculating at each rotational orientation the corresponding *V*(*c*, *p*) value. The final interaction energy *V*(*c*, *p*) for a given cage *c* and property *p* corresponds to the maximal interaction energy obtained this way. The entire process is repeated for each cage and for each atomic property, hence a typical spectrophore vector consists of *c* times *p* values, with *c* being the number of artificial cages that are used and *p* the number of different atomic properties. In the current implementation, default values for *c* and *p* are 12 and 4, respectively, meaning that 12 different cages and 4 different atomic properties are used, thereby generating spectrophores of 48 values per molecule (Fig. 3). The 48 values are organized into four sets of 12 values each:

- Values 01–12: optimal interaction energies calculated from the atomic partial charges;
- Values 13–24: optimal interaction energies calculated from the atomic lipophilicities;
- Values 25–36: optimal interaction energies calculated from the atomic shape deviations;
- Values 37–48: optimal interaction energies calculated from the atomic electrophilicities.

It should be noted that other spectrophore sizes are also possible. When the asymmetrical cages are selected (Table 1) the resulting spectrophore consists of 18 × 4 = 72 values. In case both the asymmetric and symmetric cages are selected, the resulting spectrophore will consist of 30 × 4 = 120 values. However, by default the non-stereospecific set of 12 cages are used, resulting in a spectrophore of 48 values.

### Adjustable parameters

#### Accuracy

As mentioned previously, the total interaction energy between a given cage and molecule for a given property is optimized by sampling the molecular orientation in angular steps of a given magnitude. Larger angular step sizes lead to faster computing times, but at the risk of missing the global interaction energy maximum, leading to a dependency of the spectrophore values on the actual starting orientation. Smaller angular step sizes sample the orientational space more thoroughly, but at a much higher computational cost. In our current implementation, accuracy is restricted to angular step sizes of 1°, 2°, 5°, 10°, 15°, 20°, 30°, 36°, 45° or 60° along all three axes. The user can specify this step size and therefore influence the required accuracy of the method.

#### Resolution

Spectrophores capture information about the property fields surrounding the molecule. The closer the surrounding cage is wrapped around the molecule the more atomic details and variations are captured in the resulting spectrophore values. The default distance between the molecule and cage is 3 Å, as this resembles a non-bonded average distance between the receptor and ligand. Computational time is not influenced by the applied resolution setting. In the current implementation, resolution can be specified by any real number that is larger than 0.

#### Stereospecificity

As previously mentioned, there are 12 cages that are symmetrical and 18 cages with an asymmetrical distribution of points. These latter cages are therefore sensitive to the enantiomeric configuration of the molecule within the cage. For example, the generated spectrophores of both enantiomers of a chiral molecule will be of opposite sign to each other. In most instances, the symmetric cages will suffice for normal usage of the spectrophore technology. In the current implementation, there are three stereospecificity settings: ‘none’ for no stereoselectivity (hence using the 12 symmetric cages), ‘unique’ for using only the 18 asymmetric cages, and ‘all’ for using the 12 symmetric and 18 asymmetric cages.

#### Normalization

In some circumstances it may be desirable to focus on the relative differences in the spectrophore values rather than on the absolute numbers, and for this reason normalization of the calculated values may be needed. Normalization may be important when comparing spectrophores of charged and neutral molecules, since the presence of a formal charge in the molecule will lead to a shift in the spectrophore values of the atomic charge and electrophilic properties: the lipophilicity and shape deviation spectrophore points are not influenced by the presence or absence of a formal charge. Normalization is performed on a ‘per-property’ basis, meaning that normalization is only performed on the data points belonging to the same property and not across all the data points. In our current implementation, there were four normalization settings: ‘none’ for no normalization, ‘mean’ for normalization by zero mean, ‘std’ for normalization by unit standard deviation, and ‘all’ for normalization by zero mean and unit standard deviation.

## Results and discussion

### Conformational flexibility dependency

**Fig. 4:**
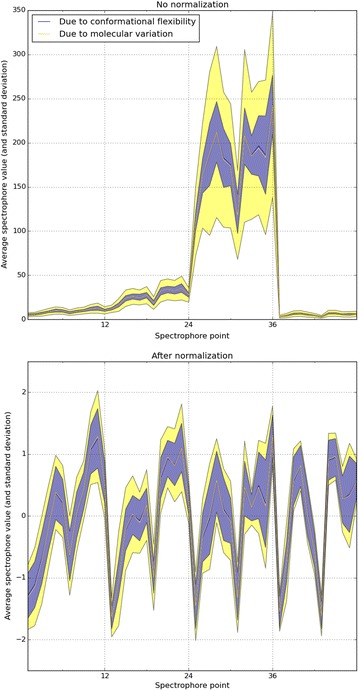
Comparison of the variations in spectrophore values resulting from molecular flexibility or molecular variability. The top figure shows the average of the normalized spectrophore values calculated across the different conformations of each molecule (yellow) or across molecules (blue). The colored areas indicate the average value ± one standard deviation. The bottom figure shows the same, but on the corresponding normalized spectrophore values. Focusing on the normalized values, the average standard deviation of the spectrophore values across conformations was 0.37, while the corresponding value across molecules was 0.57

The DUD-E dataset [45, 46] was used as a source for the selection of 1000 random compounds. Conformations were generated using RDKit [49]. For each molecule, the number of conformations generated was equal to 1.5 times the number of atoms. Each conformation was converted into a spectrophore using the default values (accuracy: 20°, resolution: 3 Å, stereospecificity: ‘none’). The standard deviation of the spectrophore values from different molecules (using only the first conformation of each molecule to calculate the spectrophore from) was compared to the standard deviation of the spectrophore values calculated from the different molecular conformations. The results are summarized in Fig. 4, and demonstrate that the variation in the spectrophore values, resulting from the conformational flexibility, is less than the variability resulting from molecular differences: hence spectrophores from different molecules show more variation than spectrophores from different conformations of the same molecule.

### Influence of the resolution and accuracy settings

**Fig. 5:**
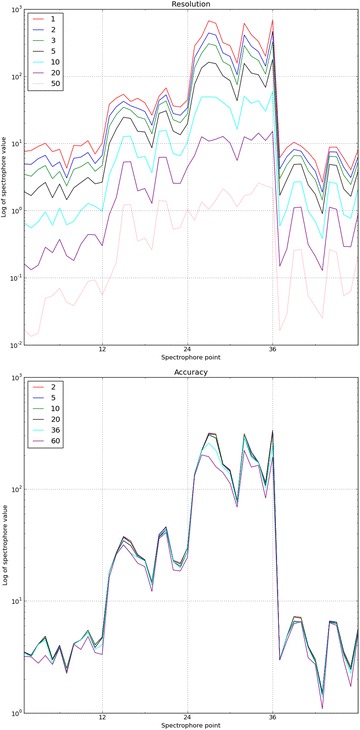
The dependency of the spectrophore values on the resolution (top) and accuracy settings (bottom). A single conformation of domperidone was used to calculate the spectrophores. All spectrophores were calculated with stereochemistry and normalization flags both set to ‘none’. To generate the top figure, accuracy was set to the default value of 20°, and for the generation of the bottom figure the resolution was set to 3 Å. Plotted along the x-axes are the 48 individual spectrophore points, and plotted along the y-axes is the logarithm of the calculated spectrophore values

The resolution of the spectrophore calculations is controlled by the distance between the central molecule and its surrounding cage. In Fig. 5 (top), the dependency of the spectrophore values on the resolution is shown. With increasing values in resolution setting, the absolute values of the calculated spectrophores become smaller, which is a consequence of the larger distance between the molecule and its surrounding cage. Changes in resolution often do not modify the overall shape of the resulting spectrophores, but minor local changes in the values are nonetheless observable (for example in the 1–12 region of the spectrophore; see Fig. 5). It is therefore important to use an identical resolution setting when comparing spectrophores of different molecules. In our current spectrophore implementation, a distance of 3 Å was used as the default resolution, since this distance reflects the typical non-bonded distance between ligand and its surrounding receptor.

Accuracy of the spectrophore calculations is specified by the angular step size that is used to rotate the molecule within its surrounding cage. Smaller step sizes can lead to significantly longer calculation times, while larger step sizes are much faster in sampling the rotational space but at the risk of missing the global interaction energy minimum. As shown in Fig. 5 (bottom), the best compromise between accuracy and computing time is obtained with an angular step size of 20°, as when using this step size there are no significant differences with the corresponding spectrophore values obtained using smaller step sizes. In contrast, accuracy settings of 36° and 60° lead to significant deviations in the calculated values.

### Application note 1: precision and recall calculated on the DUD-E dataset

The DUD-E dataset [45, 46] was used to evaluate the potential use of spectrophores in the field of virtual screening. The original DUD-E dataset contains a total of 22,886 active compounds for 102 different pharmacological targets, with each active compound ‘diluted’ by an average of 50 decoy structures. For each of the 102 pharmacological targets, a reference compound was selected and the ranked Euclidean distances between the spectrophores of these reference compounds and all other DUD-E spectrophores were used to calculate the area under the curve (AUC) from the corresponding receiver operating characteristic (ROC) curves. Spectrophores were calculated with varying normalization parameters and stereospecificity settings, however with a constant default resolution of 3 Å and a constant angular step size of 20°. Conformations were those as provided in the DUD-E dataset. The results are summarized in Table 3.

**Table 3:** Median AUC values with standard deviations calculated from the DUD-E dataset

Normalization:	Stereospecificity		
	‘None’	‘Unique’	‘All’
‘None’	0.58 ± 0.18	0.55 ± 0.19	0.55 ± 0.19
‘Mean’	0.63 ± 0.13	0.61 ± 0.13	0.62 ± 0.13
‘Std’	0.56 ± 0.14	0.57 ± 0.12	0.57 ± 0.13
‘All’	0.61 ± 0.14	0.62 ± 0.14	0.62 ± 0.14

Spectrophores were calculated with varying parameters. Firstly, spectrophore normalization was varied from no normalization at all (‘none’), normalization along the average (‘mean’) or standard deviation (‘std’), or normalization by both average and standard deviation (‘all’). Secondly, stereospecificity was either neglected using only the 12 non-stereospecific cages in the calculation of the spectrophores (stereospecificity ‘none’), included using the 18 stereospecific cages (stereospecificity ‘unique’), or using both the 12 non-stereospecific and 18 stereospecific cages (stereospecificity ‘all’)

The best median AUC value (0.63 ± 0.13) was obtained when normalization was calculated over the average (normalization ‘mean’) and with stereo option ‘none’ (hence using the 12 non-stereospecific cages). This AUC value came close to the AUC of 0.66 as calculated using Morgan fingerprints [50, 51] in combination with the Tanimoto similarity index (data not shown) [52]. On the other hand, the worst AUC median (0.55 ± 0.19) was found without normalization (normalization ‘none’) and when only the 18 stereospecific probes (stereospecificity ‘unique’) were used.

**Fig. 6:**
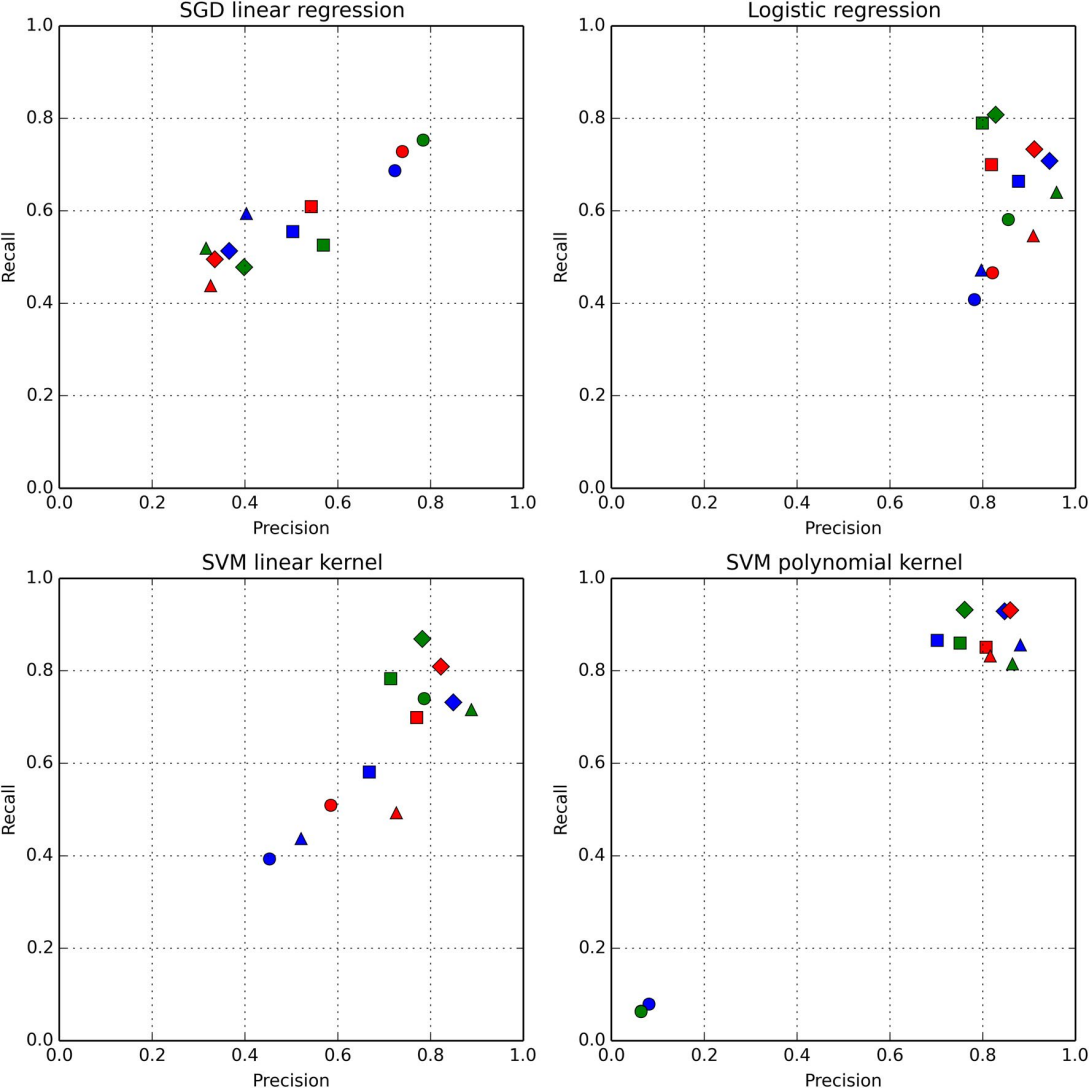
Average recall and precision parameters calculated for a number of machine learning classification methods applied to the DUD-E datasets. Recall is defined as the ratio of the retrieved true active compounds to all active compounds in the dataset, and precision is the ratio of the retrieved true active compounds to all predicted active compounds in the dataset. Normalization parameters are indicated by the marker shapes (diamond: ‘none’; square: ‘mean’; triangle-up: ‘std’; circle: ‘all’), and treatment of stereospecificity is indicated by the marker colors (blue: ‘none’; red: ‘unique’, green: ‘all’)

Spectrophores are vectors of real numbers and can therefore be used in machine learning applications to classify active from inactive compounds. The example shown in Fig. 6 applies four machine learning approaches [(a) stochastic gradient descent (SGD) linear regression, (b) logistic regression and support vector machine (SVM) with both a linear (c) and a polynomial (d) kernel], as implemented within the scikit-learn package [53], to classify active from inactive compounds within 102 targets of the original DUD-E dataset [45, 46]. For each molecule, spectrophores were calculated from the single molecular conformation as provided in the DUD-E set, each of them differing in the applied normalization and treatment of stereospecificity. Each dataset was cross-validated ten-fold using 10 stratified subsets, and the best models were selected based on maximum precision and recall. At the end, averages of the 102 recall and precision values were calculated and these averages are plotted in Fig. 6.

In general, logistic regression and SVM with a polynomial kernel gave the best results. The best values of precision were found using logistic regression with normalization and stereospecificity both set to ‘none’ (precision = 0.94 and recall = 0.71), or with normalization set to ‘std’ and stereospecificity set to ‘all’ (precision = 0.96 and recall = 0.64). The best result for recall was obtained using the SVM polynomial kernel with normalization set to ‘none’ and stereospecificity set to ‘unique’ (precision = 0.79 and recall = 0.94) (Fig. 6).

The obtained metrics are comparable to results obtained with other approaches based on 2D-fingerprints or standard shape-based methods, indicating that the spectrophore technology can also be used as a virtual screening platform. However, direct comparison between the different approaches, and in particular questions aimed at answering which method is the’best’, are in our opinion not useful since each method or approach has its own application domain and one method may be more applicable or desired compared depending on the question to be answered.

### Application note 2: scaffold hopping

**Fig. 7:**
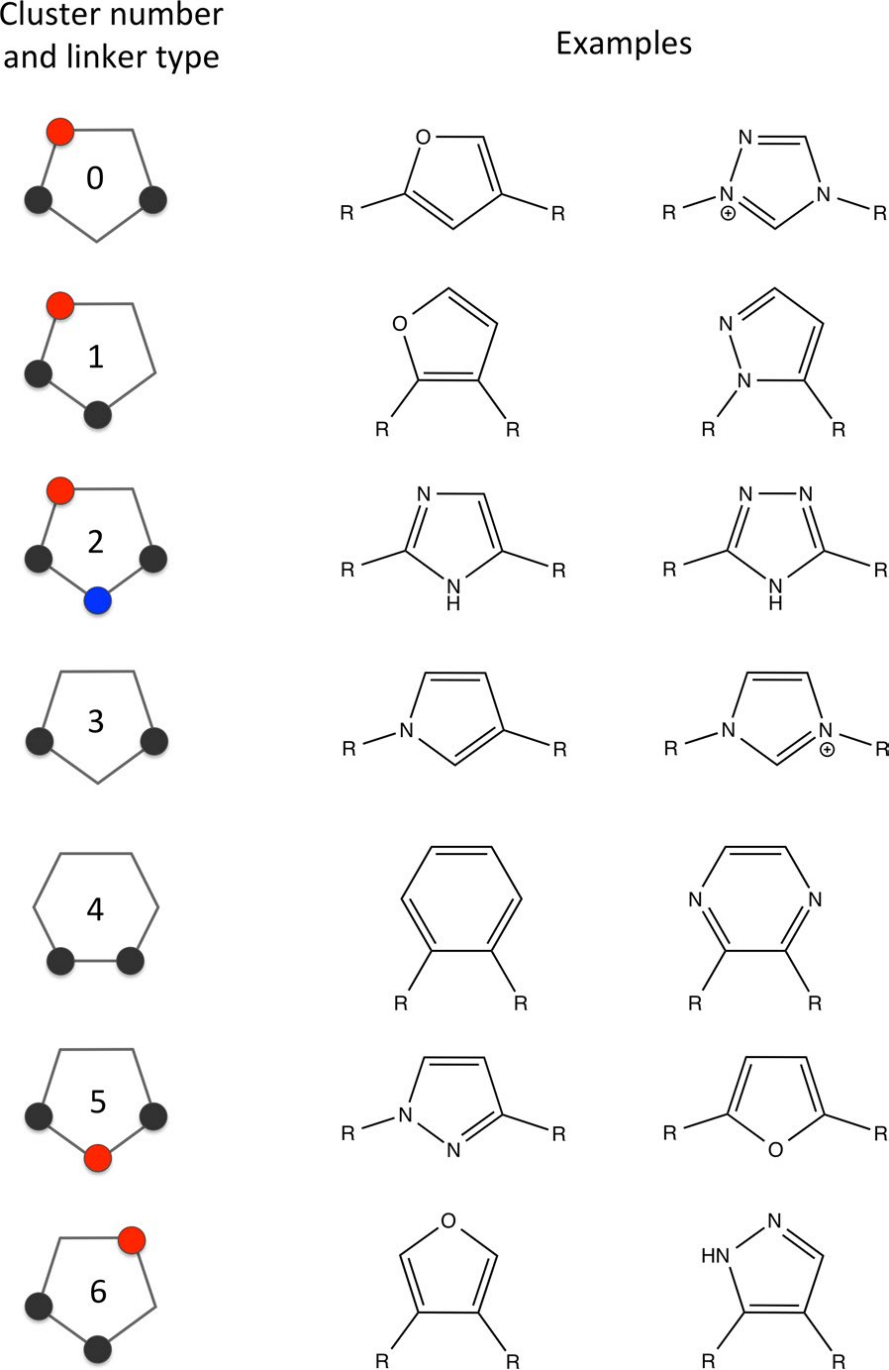
Overview of the seven identified ring templates. Black spheres indicate attachment points of the ring sidechains, red spheres indicate a hydrogen bond acceptor pharmacophore and a blue sphere indicates a hydrogen bond donor pharmacophore point. The numbers on each ring identifies the cluster number. The number of ring members in each cluster is summarized in Table 4

Spectrophores are calculated as interaction energies between a set of atomic properties and a set of artificial receptors, *in casu* a set of cages represented by 12 cage points each with a + 1 or − 1 value. With respect to this, since only the atomic properties themselves, and not the actual atom types, contribute to the final spectrophore values, spectrophores can be useful for scaffold hopping in which one wants to identify fragments with similar interaction properties but with different atomic and topological environments. In order to demonstrate the applicability of spectrophores to scaffold hopping, all five- and six-membered disubstituted aromatic rings were extracted from the DUD-E dataset [45, 46] and converted into their corresponding spectrophores (using full normalization and with only the 12 non-stereospecific cages) after replacing each of the two sidechains by a methyl group and generation of a single conformer for each ring. Subsequent clustering of the calculated spectrophores using the affinity propagation implementation of scikit-learn [53] classified the 72 different ring systems into seven different clusters, populated with 4–17 members each (Table 4; for a list of all ring systems and their corresponding cluster, see Additional file 1: S1). For the majority of clusters, a consensus chemical scaffold could be identified; these scaffolds are shown in Fig. 7. As demonstrated in Table 4 and Fig. 7, it can be seen that ring types 0, 1, 3, 5 and 6 are very well separated from the other types. Ring types 0, 3 and 5 are all 1,3-disubstituted five-membered ring systems, with a hydrogen bond acceptor functionality in the 4- and 2-position for types 0 and 5, respectively. Ring types 1 and 6 are 1,2-disubstituted five-membered rings, the difference between these two is the presence of a hydrogen bond acceptor pharmacophore at position 3 and 4 for type 1 and 6, respectively. Clusters 2 and 4 are less well clearly defined, with cluster type 2 being a mixture of rings of type 0, 1, 2, together with a significant fraction of rings which cannot be not classified into these seven ring clusters. Finally, cluster type 4 is merely composed of 1,2-disubstituted six-membered rings with a significant contamination of rings which can be classified as ring type 1 (Table 4).

**Table 4:** Cross-contamination between the seven cluster types

Cluster (linker type)^a^	Total number of rings in cluster	Number of rings of given linker type:							
		Type 0	Type 1	Type 2	Type 3	Type 4	Type 5	Type 6	Other
0	12	91.7%	–	–	–	–	8.3%	–	–
1	12	–	83.3%	–	–	8.3%	–	–	8.3%
2	10	20.0%	10.0%	20.0%	–	–	–	–	50.0%
3	4	25.0%	–	–	75.0%	–	–	–	–
4	17	–	35.3%	–	–	47.1%	–	–	17.6%
5	8	–	–	–	–	–	100.0%	–	–
6	9	–	–	–	–	–	–	88.9%	11.1%

Cluster types are defined in Fig. 7. For each cluster, the total number of different ring members as well as the number of rings of a certain cluster type are indicated. For example, of the 12 ring type members in cluster 0, eleven of these rings (91.7%) are of type 0; one ring (8.3%) is of type 5 and therefore misclassified. Ring types 0, 1, 3, 5 and 6 are best separated from the other types, while ring clusters 2 and 4 are merely a mixture of different other types

^a^See Fig. 7 for ring definitions

Again, as in the case of case study 1, we do not want make any statements whether the spectrophore approach in scaffold hopping is better than any other method; we have only included this case in order to demonstrate the applicability of the technology in scaffold hopping and the use of it in compound clustering.

### Application note 3: combining machine learning and spectrophores for the identification of novel inhibitors from compound databases

In order to demonstrate the applicability of spectrophores in the domain of virtual screening, mathematical models were generated from the spectrophores, and these models were subsequently used to identify compounds predicted to be inhibitory active against a particular subset of therapeutic targets. Following this virtual screening step, a number of these compounds were actually acquired and their predicted inhibitory activity was subsequently biochemically validated.

#### Training set

The training set was constructed from the DUD-E dataset [45, 46]. For each compound in the DUD-E set, ten conformations were generated using RDKit [49] and each conformation was converted into a spectrophore with default parameters (accuracy: 20°, resolution: 3 Å, stereospecificity: ‘none’). All ten spectrophores of each molecule were then merged into a single spectrophore by calculating the element-wise maximum of all the ten spectrophores. The training set hence consisted of 22,802 spectrophores corresponding to the ‘active’ compounds, and 1180,480 spectrophores corresponding to the ‘inactive’ compounds (the decoy set in DUD-E).

#### Building the classifiers

In the first phase of this virtual screening experiment, a binary classification machine learning model was trained and selected based on maximal ‘precision’ to classify ‘active’ spectrophores from ‘inactive’ ones (in order to limit the number of false positives, ‘precision’ was chosen as a model evaluation parameter). All models were generated using the scikit-learn package in Python [53]. The best model comprised a majority soft voting model with a random forest and *k*-nearest neighbors model as underlying classifiers (Table 5, phase 1). A second model was also generated in which the training set consisted of all ‘active’ spectrophores labeled according their DUD-E pharmacological targets, and in which only these ‘active’ compounds were used to train the particular model (hence no ‘inactives’ in this training set). This multiclass model was used in the second phase of the virtual screening experiment to assign the most likely pharmacological target label to each of the ‘active’ compounds that were selected during the first phase. The best multiclass model with ‘precision’ as evaluation parameter was the Extra Trees classifier (Table 5, phase 2). Cross-validated precision scores for this model are given in Table 6.

**Table 5:** Summary of the classifier models that have been used in the two phases of the virtual screening experiment

Phase	Classifier with parameters^a^	Precision ± SD^b^
Phase 1 (binary classification)	Soft voting classifier with 2 underlying models: Random forest classifier: criterion = ‘entropy’; max_features = ‘log2’; n_estimators = 30 k-nearest neighbors classifier: n_neighbors = 28; weights = ‘uniform’	0.80 ± 0.07
Phase 2 (multiclass)	Extra Trees classifier: max_features = None; criterion = ‘gini’; n_estimators = 90; min_samples_leaf = 1	0.63 ± 0.01

^a^Parameters as implemented in the scikit-learn package

^b^Mean and standard deviation calculated from tenfold cross-validation

**Table 6:** Results from the final screening phase in which all 93 ‘active’ compounds were labeled according their predicted pharmacological target

Target	**#**	Cross-validated precision ± SD
Acetylcholinesterase	25	0.742 ± 0.028
Tyrosine-protein kinase SRC	10	0.532 ± 0.038
Dopamine D3 receptor	9	0.668 ± 0.024
Peroxisome proliferator-activated receptor γ	5	0.384 ± 0.022
Thrombin	5	0.425 ± 0.022
Tyrosine-protein kinase LCK	4	0.570 ± 0.035
Peroxisome proliferator-activated receptor α	4	0.331 ± 0.031
ADAM17	2	0.487 ± 0.033
β-2 adrenergic receptor	2	0.520 ± 0.033
Epidermal growth factor receptor erbB1	2	0.559 ± 0.028
Protein farnesyl transferase/geranylgeranyl transferase type I α subunit	2	0.469 ± 0.022
Histone deacetylase 8	2	0.460 ± 0.037
TGF-β receptor type I	2	0.818 ± 0.043
Cyclin-dependent kinase 2	1	0.649 ± 0.041
Cytochrome P450 3A4	1	0.742 ± 0.088
Coagulation factor VII	1	0.657 ± 0.050
Focal adhesion kinase 1	1	0.661 ± 0.033
Farnesyl diphosphate synthase	1	0.980 ± 0.016
β-glucocerebrosidase	1	0.861 ± 0.082
Histone deacetylase 2	1	0.415 ± 0.048
Human immunodeficiency virus type 1 protease	1	0.435 ± 0.029
HMG-CoA reductase	1	0.640 ± 0.059
Stem cell growth factor receptor	1	0.474 ± 0.059
MAP kinase-activated protein kinase 2	1	0.805 ± 0.053
Poly [ADP-ribose] polymerase-1	1	0.597 ± 0.014
Peroxisome proliferator-activated receptor γ	1	0.467 ± 0.035
Dihydroorotate dehydrogenase	1	0.870 ± 0.042
Renin	1	0.704 ± 0.061
Retinoid X receptor α	1	0.811 ± 0.027
Trypsin I	1	0.363 ± 0.010
Tryptase β-1	1	0.627 ± 0.059
Vascular endothelial growth factor receptor 2	1	0.512 ± 0.036

The number of compounds that are assigned to each particular class is indicated (‘#’), as well as the tenfold cross-validated precision for each target as an indicator of the prediction quality for each class

#### Virtual screening of a test set

**Fig. 8:**
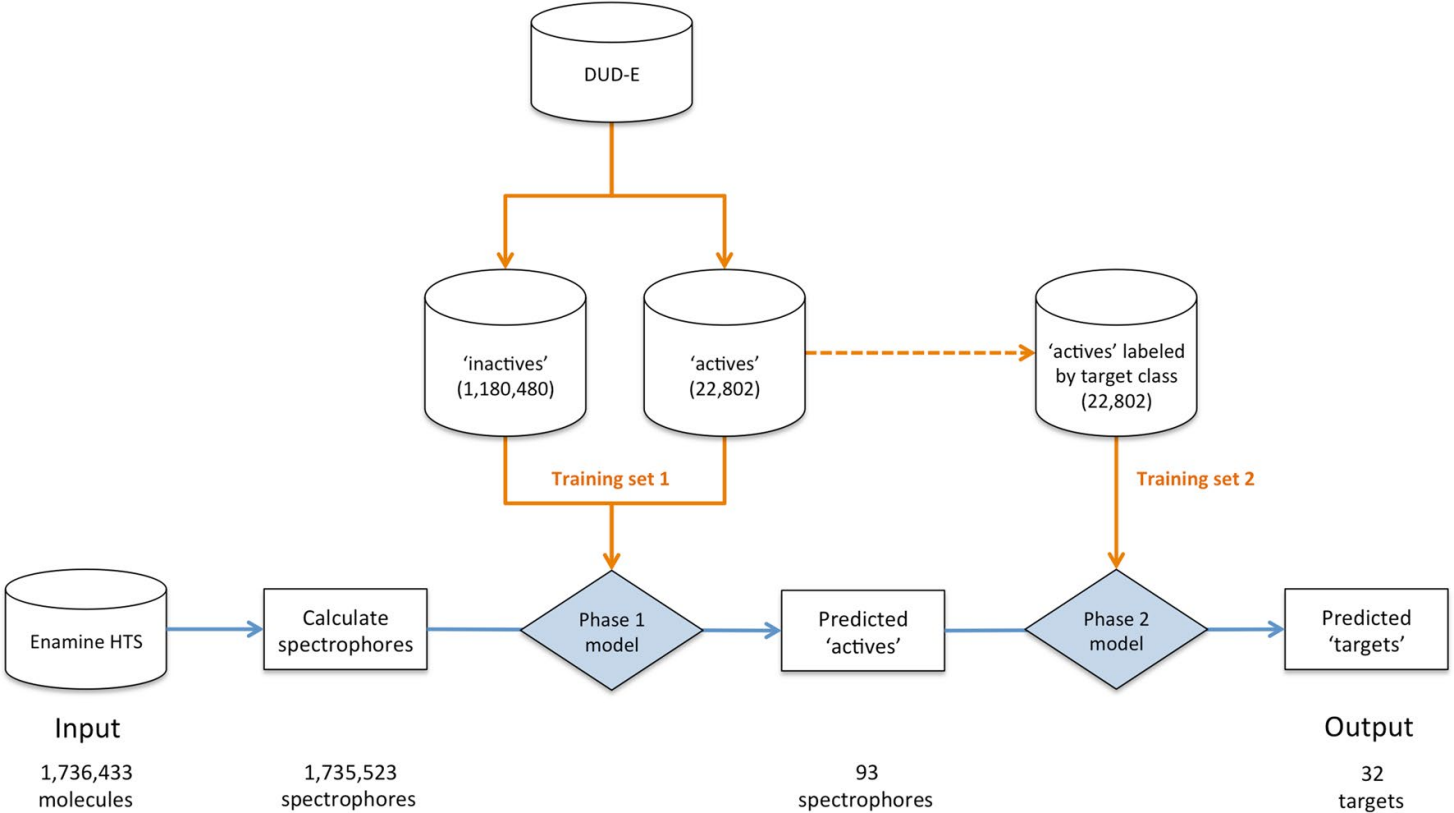
Representation of the virtual screening flow and its two phases (phase 1 and phase 2 screening). Orange arrows indicate the ‘training’ pass using the DUD-E dataset as training set, and blue arrows the ‘screening’ pass with the Enamine HTS library as input set. The generated output consists of a list of spectrophores (corresponding to Enamine molecules) labeled with their most likely pharmacological target. In total, the 93 spectrophores were assigned to 32 different labels (out of the 102 possibilities)

The Enamine HTS compound collection [54] was downloaded as a test set and the 1735,523 compounds were converted into their respective spectrophores using the same protocol as for the training set. Screening of this test set was performed in two phases (Fig. 8). In the first phase, all Enamine spectrophores were classified as either ‘active’ or ‘inactive’ according the prediction calculated by the phase 1 classifier (Fig. 8 and Table 5). In total, 93 of these spectrophores were labeled as ‘active’. In the second phase, all 93 ‘actives’ were filtered through the phase 2 classifier model in which each of these spectrophores were labeled with one of the 102 possible pharmacological classes according to the prediction made by phase 2 classifier. In total, 32 different labels were assigned to each of the 93 spectrophores. The results are summarized in Table 6 and demonstrate that 2/3 of all 93 spectrophores (read: compounds) are assigned to only seven pharmacological classes: acetylcholinesterase (25 compounds), tyrosine-protein kinase SRC (10) and LCK (4), dopamine receptor D3 (9), peroxisome proliferator-activated receptor α (4) and γ (5), and thrombin (5). The quality of these predictions (expressed as ‘precision’) ranges from ‘low’ (0.33, 0.38, and 0.42 for the peroxisome proliferator-activated receptor α and γ, and thrombin, respectively), over ‘medium’ for the two tyrosine-protein kinases SRC and LCK (0.53 and 0.57, respectively), and up to ‘good’ for the dopamine D3 receptor and acetylcholinesterase (0.67 and 0.74, respectively). The highest quality models in term of ‘precision’ are those for farnesyl transferase (0.98), β-glucocerebrosidase (0.81) and dihydroorotate dehydrogenase (0.87).

#### Validating the two highest quality models

**Fig. 9:**
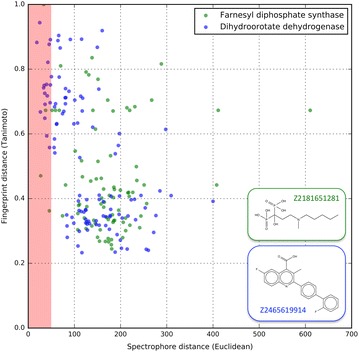
(Green dots) Calculated distances between Z2181651281 and all of the farnesyl diphosphate synthase inhibitors from the DUD-E dataset (85 compounds) expressed as spectrophores (Euclidean distance, abcis) and topological fingerprints (Tanimoto distance; ordinate). (Blue dots) Same as for the green dots, but now for Z2465619914 and all of the dihydroorotate dehydrogenase inhibitors from the DUD-E dataset (111 compounds). The red shaded area highlights all DUD-E actives that are within an Euclidean distance of 50 from Z2181651281 or Z246561914. For farnesyl diphosphate synthase (green), the are 4 such compounds (including Z2181651281), and for dihydroorotate dehydrogenase (blue) there are 17 such compounds

According Table 6, the two best models are farnesyl diphosphate synthase and dihydroorotate dehydrogenase, with tenfold cross validated specificity values of 0.980 ± 0.016 and 0.870 ± 0.042, respectively. For each of these targets only a single compound from the Enamine library was predicted to bind, and these structures with corresponding Enamine codes are given in Table 7. The calculated distances, expressed as Tanimoto and Euclidean distances, between each of the two hits and the corresponding actives from the DUD-E dataset are plotted in Fig. 9.

**Table 7:** Structure and Enamine codes of the two compounds that were predicted to hit farnesyl diphosphate synthase (left) and dihydroorotate dehydrogenase (right) according the phase 2 classification model

Farnesyl diphosphate synthase	Dihydroorotate dehydrogenase

Focusing in first instance on the farnesyl diphosphate synthase target, the hit retrieved from the Enamine HTS library for this enzyme was Z2181651281, a compound also known as ibandronic acid and described as a potent farnesyl pyrophosphate synthase inhibitor [55]. Unfortunately, this compound was also one of the 85 compounds that were part of the DUD-E training set used to derive the farnesyl diphosphate synthase model, and therefore we cannot conclude that the model has been able to identify a novel farnesyl diphosphate synthase inhibitor. However, in Fig. 9, the Euclidean and Tanimoto distances between Z2181651281 and each of the 85 DUD-E compounds are plotted (green dots). Inspection of these Euclidean distances reveal three other compounds that have a significant spectrophore similarity to Z2181651281 (Euclidean spectrophore distance ≤ 50). The structures of these three compounds, together with their topological and spectrophore distances to Z2181651281, is given in Table 8. All these compounds are bisphosphonates and are structurally very similar as reflected by the large spectrophore similarities. However, the calculated Tanimoto similarities range from 0.36 to 0.67, values which are significantly below the generally accepted cutoff of 0.85 to reflect chemical similarity [56]. Hence, these compounds would not have been retrieved as putative farnesyl diphosphate synthase inhibitors when searched with topological fingerprints and a Tanimoto similarity cutoff ≥ 0.7, while using spectrophores in combination with an Euclidean distance cutoff ≤ 50 would have retrieved all three compounds.

**Table 8:** All farnesyl diphosphate synthase and dihydroorotate dehydrogenase inhibitors from the DUD-E library with an Euclidean spectrophore distance less than 50 from their corresponding reference structures (Z2181651281 and Z2465619914, respectively)

Structure of the closest DUD-E farnesyl diphosphate synthase inhibitors	Euclidean distance to Z2181651281 (spectrophore)	Tanimoto distance to Z2181651281 (topology)
	37.8	0.673
	27.1	0.470
	45.9	0.362
	25.1	0.944
	48.1	0.891
	18.8	0.882
	45.5	0.876
	42.7	0.821
	39.9	0.753
	48.1	0.751
	34.8	0.750
	33.5	0.742
	36.0	0.725
	41.4	0.717
	47.6	0.696
	36.6	0.691
	26.5	0.668
	36.0	0.648
	40.3	0.594
	46.3	0.558

The correlation coefficient r^2^ calculated between the spectrophore (euclidean) and the topological distances (Tanimoto) is 0.027

The single compound identified as hit of dihydroorotate dehydrogenase is Z2465619914 (Table 7). This compound is also known as brequinar and has been described as a potent dihydroorotate dehydrogenase inhibitor [57], hence validating our virtual screening approach using spectrophores as similarity metric. Within the DUD-E dataset, there are 17 compounds having a spectrophore Euclidean distance less than 50 to Z2465619914 (Fig. 9). The structures of these compounds are shown in Table 8. From this table it is clear that most of these 17 compounds are quite similar to Z2465619914, however six of these compounds have a Tanimoto similarity ≤ 0.7 while their spectrophore similarity is still within the cutoff window of 50. Hence the same conclusion as for farnesyl diphosphate synthase can be made here: these compounds would not have been retrieved as putative inhibitors when searched with topological fingerprints and a Tanimoto similarity cutoff ≥ 0.7; however a different result would have been obtained using spectrophores in combination with an Euclidean distance cutoff ≤ 50.

#### In vitro biochemical validation of the predictions

In order to validate the predictions in an orthogonal manner, in vitro biochemical testing of the inhibitory activities of some of the compounds was performed. Driven by the availability of a number of *in house* biochemical assays on the one hand, but on the other hand also being limited by financial restrictions, we selected acetylcholinesterase (medium quality model, see Table 6) and thrombin (low quality classification model) as pharmacological targets for the biochemical validation. As shown in Table 6, five compounds were predicted to target thrombin and 25 compounds targeted acetylcholinesterase; hence these 30 compounds were ordered from Enamine in 5 mg solid state quantities each. All 30 purchased compounds were tested in both biochemical assays in concentrations ranging from 100 to 1 μM. Acetylcholinesterase activity was determined by a kinetic assay using the indicator 5,5′-dithiobis-(2-nitrobenzoic acid) (DTNB, Ellman’s reagent) and the substrate acetylthiocholine iodide (*K*_*m*_ = 420 μM) at concentration 500 and 400 μM, respectively. All the experiments were conducted in duplicate at 25 °C in a 100 mM phosphate buffer at pH 7.8. Control experiment using a commercial inhibitor of acetylcholinesterase, neostigmine methyl sulfate (IC_50_ ≈ 40 nM), was included in each screening assay. Thrombin activity was determined by a kinetic assay using the chromogenic substrate Biophen CS-21(66) (pyro-Glu-Pro-Arg-pNA∙HCl, *K*_m_ = 400 μM) at 415 μM concentration. All the experiments were conducted in duplicate at 37 °C in 50 mM HEPES buffer at pH 8.1. Control experiment using a commercial inhibitor of thrombin, gabexate mesylate (*K*_i_ = 500 nM) was included in each screening assay. Detailed protocols for the biochemical assays are described in Additional file 2: S2.

The results obtained from the biochemical assays are summarized in Table 9. Protocols used for the biochemical assays are described in Additional file 2: S2. Many of the compounds were difficult to solubilize in the assay buffers, so the highest concentration at which measurements could be done was determined by the actual compound solubility. Additionally, acetylcholinesterase is very sensitive to the presence of DMSO, therefore the final test mixture contained 0.25% of DMSO, limiting range of the compound concentrations tested to low-micromolar values. In case of thrombin, the enzyme can tolerate better the presence of DMSO; the final percentage of DMSO in the test mixture was 2.5%, allowing to test the compounds at slightly higher concentrations. Focusing in first instance on the results obtained for the low-quality target thrombin, none of the five compounds that were predicted to target thrombin actually inhibited this enzyme at concentrations lower than 100 μM. Although this may seem disappointing at first sight, one should bear in mind that the phase 2 classification model for thrombin is of very poor quality (cross-validated precision of 0.425 ± 0.022; see Table 6) and that only five compounds were predicted to bind this pharmacological target. However, the results were far more encouraging for acetylcholinesterase. The phase 2 classification model for acetylcholinesterase was of much higher quality in terms of cross validated precision (0.742 ± 0.028; Table 6), and this is reflected in the fact that 2 out of the 25 predicted compounds (Z44853616 and Z1723688652) from the Enamine library showed inhibitory activity against this target at IC_50_ values lower than 1 μM (Table 9).

**Table 9:** Chemical structures, Enamine codes, and inhibition percentages at given concentrations of the 30 compounds tested against both the acetylcholinesterase and thrombin assays

Enamine code and structure	Predicted target^a^	Acetylcholinesterase assay/compound concentration (μM)				Thrombin assay/compound concentration (μM)			
		25	10	2.5	1	100	50	25	10
	A	48%	–	3%	–	0%	–	–	0%
	A	31%	–	0%	–	–	4%	–	0%
	A	–	–	0%^b^	–	–	–	0%	0%
	A	–	21%	0%	–	–	–	0%	0%
	A	–	27%	0%	–	–	–	6%	5%
	A	–	–	1%^b^	–	–	–	0%	0%
	A	–	18%	0%	–	–	–	0%	0%
	A	–	0%	0%	–	0%	–	–	0%
	A	–	25% (5 μM)	8%	–	–	–	–	0%
	A	–	0%	0%	–	–	0%	–	0%
	A	–	–	75%	60%	–	–	0%	0%
	A	–	22%	7%	–	0%	–	–	0%
	A	–	4%	1%	–	–	0%	–	0%
	A	–	41%	–	14%	–	–	0%	0%
	A	–	44%	–	14%	9%	2%	–	0%
	A	–	1%	2%	–	–	0%	–	0%
	A	–	8% (5 μM)	0%	–	–	0%	–	0%
	A	–	0%	0%	–	4%	–	–	0%
	A	–	0%	0%	–	0%	0%	–	0%
	A	–	11%	–	–	–	–	0%	0%
	A	–	–	82%	51% (0.25 μM)	–	–	0%	0%
	A	–	31%	16%	–	0%	0%	–	0
	A	–	32%	11%	–	0%	0%	–	0%
	A	–	10%	–	–	–	–	–	0%
	A	–	35%	7%	–	–	6%	–	0%
	T	–	48%	19%	–	–	7%	–	0%
	T	–	31%	8%	–	0%	–	–	0%
	T	–	23%	0%	–	–	0%	–	0%
	T	–	0%	–	–	–	4%	–	0%
	T	–	0%	–	–	0%	0%	–	0%

For each compound, the highest concentration for measurement was determined by the solubility of the compound

^a^Predicted target: ‘A’ stands for acetylcholinesterase, ‘T’ for thrombin

^b^These compounds could not be tested at higher concentrations due to poor solubility in the acetylcholinesterase buffer solution

**Fig. 10:**
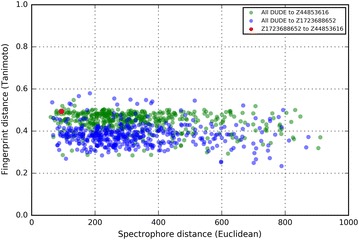
Comparison between the spectrophore and topological distances calculated between the hits and all of the acetylcholinesterase inhibitors from the DUD-E dataset (453 compounds). Green shaded dots are the distances calculated between Z44853616 and the 453 DUD-E inhibitors, and blue shaded dots are the corresponding distances calculated for Z1723688652. The red dot indicates the calculated distances between Z44853616 and Z1723688652

The two identified acetylcholinesterase inhibitors are topologically quite diverse but are very similar in spectrophore space. As shown in Fig. 10, the Tanimoto distance between the topological fingerprints generated from both compounds is 0.49 (calculated with RDKit [59]), a value far below the 0.85 cutoff that is commonly used to reflect similar bioactivities [56]. However, the Euclidean distance between the spectrophores of both compounds is on the lower edge of the spectrum (Fig. 10; red dot), indicating high similarity from a spectrophore’s point-of-view. In addition, none of the two identified compounds possess any significant topological similarity to any of the 453 acetylcholinesterase inhibitors from the DUD-E dataset (all Tanimoto distance are less than 0.6), indicating that these two compounds would likely not have been identified from the DUD-E dataset when these topology-based fingerprints would have used as similarity criterion. The lack of correlation between the Spectrophore and topological fingerprint distances is also clear from Fig. 10. The calculated squared correlation coefficients *r*^*2*^ are 0.043 and 0.004 for Z44853616 and Z1723688652, respectively.

### Implementation

The current spectrophore algorithm has been implemented in three separate frameworks: 1) an Open Babel implementation written in C++ (OBSpectrophore [58, 59]), 2) an RDKit version written in Python and C++, and 3) an RDKit implementation coded entirely in Python. On a personal computer equipped with an i7-5500U processor with 12 GB of main memory, the Open Babel version runs fastest, with an average calculation speed of 36 ± 2 ms/molecule. On the other hand, the code entirely written in Python performs the same calculations 140 times slower (4950 ± 79 ms/molecule). The mixed Python/C ++ implementation in RDKit is only 3 times slower than OBSpectrophore, performing the same calculations at an average speed of 102 ± 2 ms/molecule.

## Conclusions

The spectrophore is a novel descriptor that reflects, in a virtual manner, the way how molecules are binding to a set of artificial receptors, taking into account the spatial interactions between a molecule and its surroundings. Because of these unique properties, the spectrophore can be considered to be a one-dimensional mathematical description of a three-dimensional pharmacophore. This makes it applicable for a wide range of cheminformatics approaches, including virtual screening using sophisticated statistical models and clustering approaches. Successful applications in the area of scaffold hopping and virtual screening have been demonstrated in this study.

In the multi-target virtual screening experiment, all compounds were treated as neutral and they were not ionized according to their physiological pH. This could be one of the factors explaining the poor model quality of the thrombin target (Table 6), as it has been demonstrated that many of the thrombin inhibitors carry a positively charged functional group as a common feature binding into the P1 pocket of thrombin [60]. Although the current setup was sufficient for our novel proof-of-concept study such as this work, in a real-world virtual screening experiment the correct pretreatment and washing conditions for each compound would need to be carefully determined [61].

The spectrophore technology is one of the many existing descriptors that may be used in the field of cheminformatics and could be useful to compare some of these. However, we believe that descriptor comparison, by evaluating their performance in clustering and virtual screening, is a difficult and very subjective task as 2D- and 3D-descriptors are fundamentally different in the way they represent molecules. Therefore, this will lead to fundamental differences in the outcome of a virtual screening experiment. Rather than comparing these technologies in an attempt to identify the ‘most powerful one’ (by whichever criterion is selected), we believe that it would be more useful to integrate many of these orthogonal molecular representations into a unified machine learning model, with the goal of developing a virtual screening toolbox with optimized predictive power. Research into such an approach is currently ongoing in our laboratory.

The spectrophore technology could also be extended to describe and compare protein pockets. In this case, the spectrophore approach needs to be modified in such way that the protein pockets, along with their structural and electronic properties, are converted into a type of mirror image from which the corresponding spectrophore can be calculated. Research is currently also on-going to investigate the feasibility and applicability of this approach.

## Additional files

**Additional file 1.** Ring fragments and their corresponding clusters.

**Additional file 2.** Experimental procedures for the biochemical assay.

## Abbreviations

AUC: area under curve
QSAR: quantitative structure–activity relations
SVM: support vector machine
