# Supplementary material for: Spectrophores as one-dimensional descriptors calculated from three-dimensional atomic properties: applications ranging from scaffold hopping to multi-target virtual screening
Source: J Cheminform. 2018 Mar 7;10:9. doi: 10.1186/s13321-018-0268-9 (PMC5842169; doi:10.1186/s13321-018-0268-9)

**Supplementary material 2**

Figure S1. Ring fragments and their corresponding clusters. Indicated cluster numbers should be extracted by 1 in order to obtained the corresponding cluster number in the manuscript (for example, 5 becomes 4 in the manuscript).


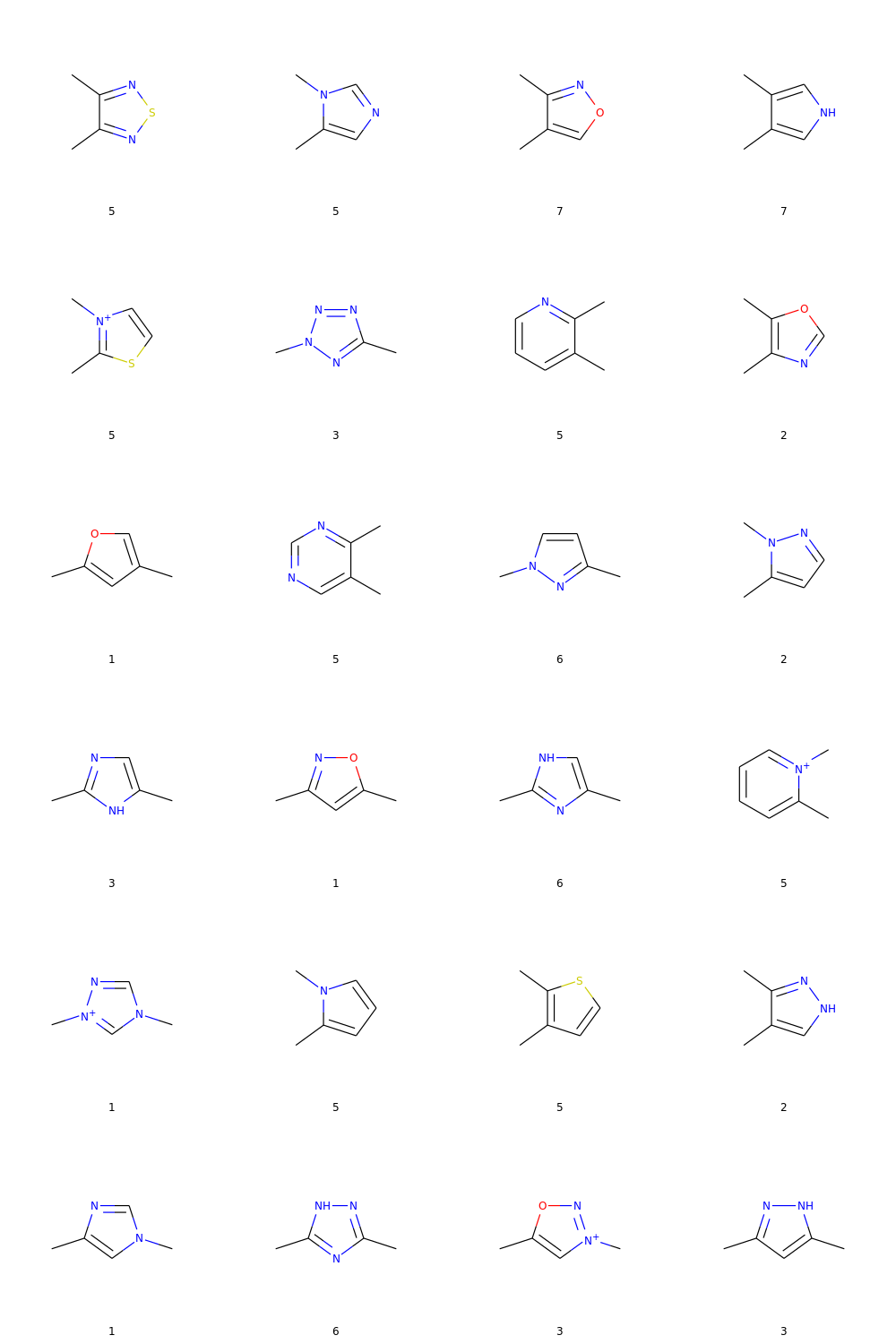


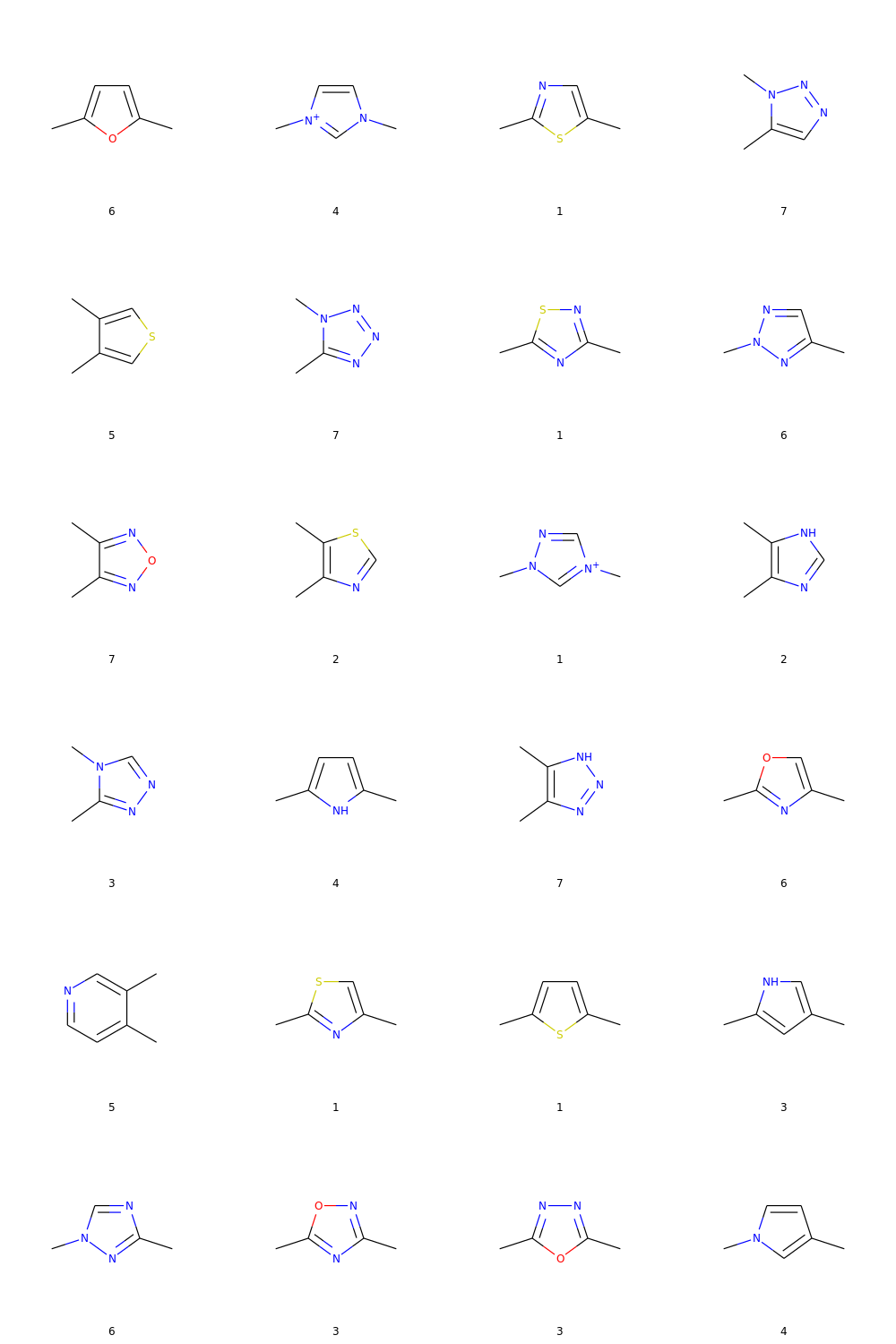


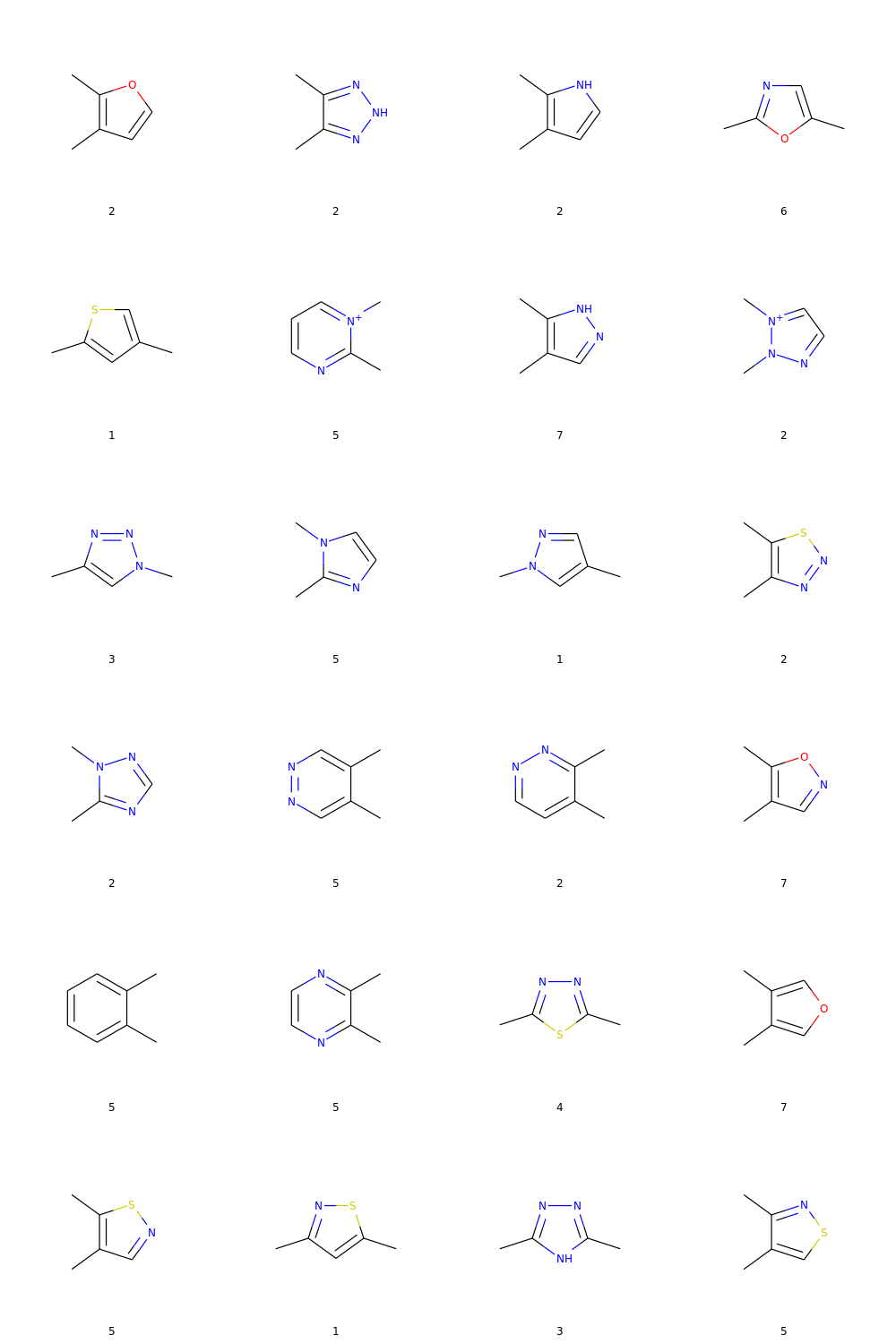

Supplement: Supplementary file 1 — Additional file 1. Ring fragments and their corresponding clusters. [file 13321_2018_268_MOESM1_ESM.docx]
