# Supplementary material for: Spectrophores as one-dimensional descriptors calculated from three-dimensional atomic properties: applications ranging from scaffold hopping to multi-target virtual screening
Source: J Cheminform. 2018 Mar 7;10:9. doi: 10.1186/s13321-018-0268-9 (PMC5842169; doi:10.1186/s13321-018-0268-9)
Supplement: Supplementary file 2 — Additional file 2. Experimental procedures for the biochemical assay. [file 13321_2018_268_MOESM2_ESM.docx]

**Supplementary material 3: biochemistry**

Enzymatic assays were performed with use of BioTek microplate reader (Synergy MX). Data collection and analysis were performed using Gen5Microplate Software and Microsoft Excel. Collection of screening compounds was purchased from Enamine and evaluated for the inhibitory activity against thrombin and acetylcholinesterase. The percentage of enzyme inhibition was determined using a spectrophotometric assay.

Thrombin (from human plasma) was obtained from Sigma-Aldrich. Inhibitor kinetic assays were using the chromogenic substrate Biophen CS-21(66) (pyro-Glu-Pro-Arg-pNA∙HCl, K_m_ = 400 μM) from HYPHEN BioMed at 415 μM concentration. All the experiments were conducted in duplicate in a 50 mM HEPES buffer (Sigma-Aldrich) at pH 8.1. The readout consisted of the evaluation of thrombin-mediated para-nitroaniline release from the chromogenic substrate pyro-Glu-Pro-Arg-pNA at given concentration (usually between 1–100 μM, depending on the compound’s solubility). Enzymatic activity was measured during 5 min at 37 °C. Absorbance was monitored at λ = 405 nM. Additionally, control experiment using commercial inhibitor of thrombin, gabexate mesylate (Enzo Life Sciences, K_i_ = 500 nM) was included in each screening assay, and showed 93% inhibition at 10 μM and 62% inhibition at 1 μM in our assay.

(Reference: Gladysz, R.; Adriaenssens, Y.; De Winter, H., Joossens, J.; Lambeir, A.-M.; Augustyns, K.; Van der Veken, P. Discovery and SAR of novel and selective inhibitors of urokinase plasminogen activator (uPA) with an imidazo[1,2-a]pyridine scaffold, Journal of Medicinal Chemistry, **2015**, 58, 9238-9257)

Human recombinant enzyme, acetylcholinesterase, was obtained from Sigma-Aldrich. Inhibitor kinetic assays were using the indicator 5,5′-dithio-bis-(2-nitrobenzoic acid) (DTNB, Ellman’s reagent) and the substrate acetylthiocholine iodide from Sigma-Aldrich. Concentrations of the indicator and the acetylthiocholine substrate (K_m_ = 420 μM) in assay were 500 μM and 400 μM, respectively. All the experiments were conducted in duplicate in a 100 mM phosphate buffer at pH 7.8. The readout consisted of the evaluation of acetylcholinesterase-mediated hydrolysis of acetylthiocholine to form thiocholine, followed by the reaction between thiocholine and DTNB to form colorful thionitrobenzoate anions. Absorbance was monitored at λ = 412 nM. The compounds were screened at concentrations in 0,25 – 25 μM range. Enzymatic activity was measured during 5 min at 25 °C. Additionally, control experiment using commercial inhibitor of acetylcholinesterase, neostigmine methyl sulfate (Sigma-Aldrich, IC_50_ ≈ 40 nM) was included in each screening assay and showed 95% inhibition at 0.25 μM and 64% inhibition at a concentration of 0.05 μM in our assay.

(Reference: Encyclopedia of Surface and Colloid Science, Volume 5, p. 3249-3250, Edited by P. Somasundaran, New York, 2006.)
